# A realist review of interventions and strategies to promote evidence-informed healthcare: a focus on change agency

**DOI:** 10.1186/1748-5908-8-107

**Published:** 2013-09-08

**Authors:** Brendan McCormack, Joanne Rycroft-Malone, Kara DeCorby, Alison M Hutchinson, Tracey Bucknall, Bridie Kent, Alyce Schultz, Erna Snelgrove-Clarke, Cheyl Stetler, Marita Titler, Lars Wallin, Valerie Wilson

**Affiliations:** 1 Institute of Nursing and Health Research/School of Nursing, University of Ulster, Shore Road, Newtownabbey, BT37 0QB, Antrim, Co, Northern Ireland; 2 School of Healthcare Sciences, Bangor University, Fron Heulog, Ffriddoedd Road, Bangor, UK; 3 School of Nursing, McMaster University, 1280 Main St W, Ontario, Hamilton, Canada; 4 School of Nursing and Midwifery, Deakin University, 221 Burwood Hwy, Victoria 3125, Burwood, Australia; 5 Deakin-Southern Health Nursing Research Centre, I Block 246 Clayton Road, Victoria 3168, Clayton, Australia; 6 Alfred-Deakin Centre for Nursing Research, The Alfred Hospital, 55 Commercial Rd, Victoria 3004, Melbourne, Australia; 7 School of Nursing and Midwifery, University of Plymouth, Drake Circus, PL4 8AA, Plymouth, UK; 8 Alyce A Schultz, Independent Consultant, 3172 Hillcrest Drive 59715, Bozeman, MT, USA; 9 School of Nursing, Dalhousie University, Nova Scotia B3H 4R2, Halifax, Canada; 10 EBP/Evaluation Consultant, 321 Middle Street Amherst, Massachusetts, & Health Services Department, Boston University School of Public Health, Boston, Massachusetts, USA; 11 School of Nursing, University of Michigan, 400 North Ingalls Building, Michigan 48109–5482, Ann Arbor, MI, USA; 12 Department of Neurobiology, Care Sciences and Society, Division of Nursing, Karolinska Institutet, Sweden; 13 Faculty of Nursing, Midwifery and Health, University of Technology Sydney, Building 10, 235-253 Jones Street, Ultimo, Australia

**Keywords:** Realist synthesis, Evidence-informed health care, Change agency, Facilitators, Opinion leaders, Knowledge brokers’ knowledge utilization

## Abstract

**Background:**

*Change agency* in its various forms is one intervention aimed at improving the effectiveness of the uptake of evidence. Facilitators, knowledge brokers and opinion leaders are examples of change agency strategies used to promote knowledge utilization. This review adopts a realist approach and addresses the following question: *What change agency characteristics work, for whom do they work, in what circumstances and why?*

**Methods:**

The literature reviewed spanned the period 1997-2007. Change agency was operationalized as roles that are aimed at effecting successful change in individuals and organizations. A theoretical framework, developed through stakeholder consultation formed the basis for a search for relevant literature. Team members, working in sub groups, independently themed the data and developed chains of inference to form a series of hypotheses regarding change agency and the role of change agency in knowledge use.

**Results:**

24, 478 electronic references were initially returned from search strategies. Preliminary screening of the article titles reduced the list of potentially relevant papers to 196. A review of full document versions of potentially relevant papers resulted in a final list of 52 papers. The findings add to the knowledge of change agency as they raise issues pertaining to how change agents’ function, how individual change agent characteristics effect evidence-informed health care, the influence of interaction between the change agent and the setting and the overall effect of change agency on knowledge utilization. Particular issues are raised such as how accessibility of the change agent, their cultural compatibility and their attitude mediate overall effectiveness. Findings also indicate the importance of promoting reflection on practice and role modeling. The findings of this study are limited by the complexity and diversity of the change agency literature, poor indexing of literature and a lack of theory-driven approaches.

**Conclusion:**

This is the first realist review of change agency. Though effectiveness evidence is weak, change agent roles are evolving, as is the literature, which requires more detailed description of interventions, outcomes measures, the context, intensity, and levels at which interventions are implemented in order to understand how change agent interventions effect evidence-informed health care.

## Background

The field of evidence-informed healthcare is broad and encompasses a variety of theories, methodologies, methods and tools. A decision maker needs to negotiate a range of interrelated cognitive, social and creative processes of evidence selection and construction, and a range of contextual factors and behavioral changes in order to make effective health care decisions [1,2]. This fact highlights the need to understand the relationships between such processes. Evidence-informed healthcare has become recognized as fundamental to practice [3,4] and aims to address the large gap between what is known and what is consistently done [5]. Evidence-informed healthcare comprises the use of the best available (least biased and most trustworthy) evidence in decision making [4] in order to ensure ethical and accountable practice [3], protect patients from incompetence and other risks [4], and achieve the best patient outcomes through organizations meeting their responsibilities for the delivery of high quality care.

Unfortunately, practitioners and policymakers have largely afforded only secondary importance to the use of evidence reviews in the implementation of healthcare interventions [6,7]. Fundamental questions still exist about which strategies should be used in particular settings [5]. Despite this limited evidence base on implementation strategies, decision makers need to determine how best to implement interventions and identify key related components that must not be compromised or adapted. As a result, there has been a call for the inclusion of broader types of evidence than have traditionally featured in clinical medicine and in evidence reviews [6]. Evidence-informed decision making models advocate for research evidence to be considered in conjunction with clinical expertise, patient preferences and values, and available resources [8]. Concerns have been expressed that systematic reviews fail to reflect the real-world interaction between evidence and action [6,9], including the diversity of disciplines, complexity and quality of analysis, and complex relationships between healthcare interventions and outcomes. Thus, despite the strong potential for reviews to inform decision making, Grimshaw *et al*. [6] highlight that they may have limited relevance or applicability, and create potentially inappropriate overreliance on their findings that could discourage innovation.

Systematic reviews typically focus on the minimization of bias, often at the expense of the details that relate to the complexity and context of interventions, which become detached from the findings and are then in danger of being overly simplified and even misleading [10,11]. Realist inquiry avoids this danger by taking an explanatory approach that examines the mechanisms of how programs work, without assuming that future interventions would work in precisely the same way as those reviewed [10]. Realist syntheses were developed in response to the weaknesses of systematic reviews and feature similar steps, with a focus on the refinement of theory related to how interventions work, rather than comparing the effectiveness of interventions [12]. Furthermore, the emphasis is on understanding how the contexts in which interventions are implemented affect the outcomes they achieve. A realist review is undertaken systematically in order to address issues of effectiveness, with the processes undertaken being similar to that of a systematic review, *i*.*e*., a comprehensive search, screening for relevance and quality in a transparent manner, and data synthesis, in order to generate findings.

Numerous systematic reviews have evaluated the effectiveness of interventions in promoting evidence-informed healthcare [13-17]. In their overview of concepts and evidence to guide knowledge translation activities, Grimshaw and colleagues highlight a multiplicity of approaches (including education and training, reminders, decision-support, local opinion leaders, and audit and feedback) that have been used individually, or in combination, to facilitate evidence use. Application of these interventions is usually accompanied by an implementation strategy, designed to support and promote the success of the intervention. An overview of 54 reviews of individual interventions or combinations of interventions, including those related to change agency [18] found that there were mixed effects for educational interventions, conferences or courses, opinion leaders, education, performance feedback, and patient-mediated interventions. In the context of change agency in evidence-informed healthcare, no systematic reviews have been undertaken. This in part may be due to the complexity of the term itself, the lack of precision in defining the term, and the multiplicity of associated terms. For example, in a comprehensive review of the diffusion of innovations in service organizations, Greenhalgh *et al*. [2] identified a range of strategies for enabling evidence use in practice, but at no stage used the term ‘change agency’. This lack of specificity and precision was reinforced in a conceptual analysis of key concepts used in knowledge transfer by Thompson and colleagues [19], who concluded that considerable confusion exists in the use of terms such as opinion leaders, facilitators, champions, linking agents, and change agents.

Thus, while numerous systematic reviews have been conducted to determine the effectiveness of specific interventions that can be associated with change agency or practices in which change agents engage, reviewers are yet to undertake a systematic review of the literature to examine the mechanisms or how such interventions work, and under what circumstances. This review fills this gap.

In this paper, we describe the results from the first stage of a study, using a realist synthesis approach, within a program of research (The ReS-IS [Realist Synthesis of Implementation Strategies] Project) that aimed to answer the question ‘what interventions and strategies are effective in enabling evidence-informed healthcare?’ A key stage in the realist synthesis methodology is ‘theory development’ , *i*.*e*., the development of a theoretical model to explain relationships. In this study, early theory development work identified four theory areas related to interventions and strategies to achieve evidence-informed healthcare, and these themes form the theoretical framework of the review. The theory development process has been described in detail in a previous publication outlining the methodology of the ReS-IS study [20]. While the ReS-IS framework encompassed four theory areas, this paper focuses on one of these – change agency, where ‘change agency’ is defined as ‘organization or other unit that promotes and supports adoption and implementation of innovations’ [6,21]. Set within this definition of change agency, we were particularly interested in the roles used to bring about change. We focused on change agency due to the sizeable body of published literature pertaining to roles that focus on bringing about change (see Rycroft-Malone *et al*. 2012 for details [20]), but as we will show later in the paper, the focus has not been on change agency per se, but on specific interventions undertaken by change agents operating in a variety of roles that relate to change agency. Further, in the context of this review, starting with change agency from the theoretical framework yielded the potential to produce meaningful results that could also inform the other theory areas within the framework.

### Review purpose

The purpose of this realist review was to determine how change agency ‘interventions’ may operate in different contexts and with what effects. This realist review is set within the standard realist evaluation or review question; *i*.*e*., ‘what works (how particular interventions [known as mechanisms] perform), for whom does it work (different individuals or populations), in what circumstances (different characteristics), and why (explanations of relationships between mechanisms and contextual characteristics)’ The overarching question in this study, therefore, was: What change agency characteristics work, for whom do they work, in what circumstances, and why? To answer this overarching question, we devised three specific questions:

1. How do the characteristics of the change agent affect knowledge utilization?

2. How does the interaction between the change agent and the setting affect knowledge utilization?

3. What is the overall effect of the change agent on knowledge utilization?

## Method

Realist synthesis [11] focuses on the study of the evidence underpinning complex interventions, particularly when the evidence base is heterogeneous and not conducive to systematic review methods [11,22]. Realist work places significance on the context and postulates that contextual influences are mobilized by the choices that human beings make, that it is possible to identify patterns in these choices (‘demi-regularities’), and that these patterns act as ‘theories’ [10,11,23]. Thus, realist synthesis is a theory-driven method and iterative process aimed at uncovering the theories that inform decisions and actions. In this review, we followed a process, which is now detailed in published reporting standards of realist reviews [24].

### Identifying initial program theories

Initial program theories were identified through an iterative process of workshops, telephone-conferences and ‘blog discussions’ by a team who were immersed in relevant literature. The team comprised 11 international knowledge translation researchers and practitioners who self-selected to participate in a working group, the aim of which was to explore the effectiveness of interventions to promote evidence-informed healthcare. Following in-depth discussion and an initial scope of the literature, four theory areas were identified for scrutiny: agency (person, roles); systems change (group or social processes); technology (mechanisms); education and learning strategies. The research team considered what quantity of an intervention is needed (dose), the target of an intervention, *e*.*g*., individual, team, organization (level), and evidence of particular contextual issues shaping the intervention (contextual factors) [20]. In this first review, we focused on ‘change agency’ , due to the sizeable body of published literature pertaining to this theory area and thus its potential to produce meaningful results that could also inform the other theory areas of the theoretical model as the review progressed.

### Searching process

The literature search was purposive in order to scrutinize the initial program theories. Search terms were compiled by the team as a list of knowledge utilization and change agency terms. The terms of reference, in conjunction with relevant indexing terms according to database, were used to guide the searches. Two team members conducted the searches of six online databases: Medline, CINAHL, Embase, PsycInfo, Sociological Abstracts, and Web of Science. Health Sciences Librarians (Dalhousie University, Halifax, Canada; McMaster University, Hamilton, Canada) were consulted in the process of constructing the search. Consistent with the purpose of the review, the search strategies were deliberately broad and did not include discipline-related terms, with one exception. In CINAHL, the indexing term ‘nursing knowledge’ was combined, using the Boolean operator ‘OR’ , with the term ‘knowledge’ , in order to capture all papers indexed using either ‘knowledge’ or ‘nursing knowledge’; however, this specific term being combined with ‘or’ along with the general term ‘knowledge’ ensured that search results were not limited only to ‘nursing knowledge’ Despite the search strategy omitting discipline-specific terms, the second level of screening, examining the full-text articles, revealed that the relevant articles returned from the search were primarily nursing-related. This result may be a consequence of publication activity being concentrated in the nursing discipline, better indexing procedures for the discipline, or intervention work primarily being conducted by nurses and reported in nursing journals that are well-indexed in the databases of published literature related to healthcare.

The searches were executed in OVID in March 2007 for the period of 1997 to 2007. Given the state of the field, searches were limited to the previous 10 years, which was considered an appropriate timeframe in the search for intervention studies in knowledge utilization. As a quality measure, one group member developed a list of 14 journals prominent in the field of knowledge utilization. A second group member reviewed the list against the indexing of the databases searched, and determined that these journals were adequately indexed in the databases selected for the search. Additionally, using their knowledge of the literature, all team members reviewed the final reference list to ensure that potential relevant papers were not missed by the search strategy.

A list of terms to refer to change agents was developed and incorporated into search strategies. The list included: Opinion leader; Facilitator/ion; Education outreach worker; Academic detailer; Practice developer; Clinical Educator; Change agent; Knowledge broker; Champion; Innovator; Boundary spanner; Advocate; Expert; Transformational leader; Consultant; Coach; Educator; Nurse researcher; EBP champion; Staff developer; Professional practice developer. Terms were joined with the Boolean operator ‘OR’ in order to capture any instance of any term’s use. Several terms were truncated in order to capture different uses of that term (*e*.*g*., facilitat*, change agen*). Where indexing terms existed already in reference to a particular concept, the indexing term was used as well as possible variations captured by keywords. Given that searches were run in March 2007, it is possible that since searches were conducted, new indexing terms have been added; however, at the time the searches were executed, the combination of indexing term and keyword use was intended to maximize sensitivity of the searches. Details of the databases and search strategy are available in Additional file 1 and further detail of the methodology can be found in previous publications [20,25].

### Selection and appraisal of documents

Search results were saved as text files and downloaded into Reference Manager Professional Version 11.0, a bibliographic software manager program. The content of the file was then backed-up to a secure server. A total of 24, 478 electronic references were returned from the change agency search strategies. Preliminary screening of the article titles reduced the list of potentially relevant papers to 196. The preliminary screen was intentionally inclusive to capture all articles potentially relevant to the review purpose of addressing what change agent interventions worked, for whom, in what circumstances, in what respects, and why. Therefore, in the interests of a comprehensive review, the initial level of screening erred on the side of inclusion wherever a title appeared to be potentially relevant to the change agent/agency concept or any of the search terms/definitions in relation to the change agent component of the theoretical framework.

**Figure 1:**
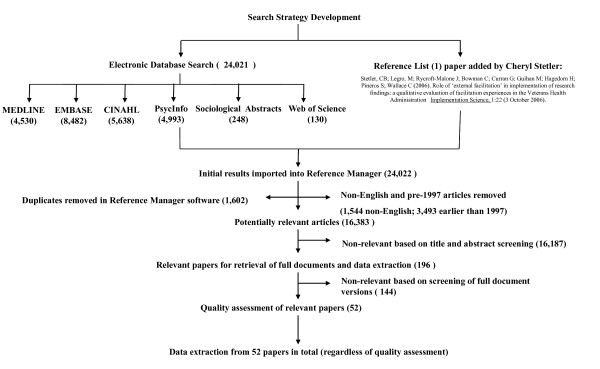
Search strategy and data extraction processes.

At this stage, all seemingly relevant papers were retrieved in full-text for a more detailed relevance test. McKibbon’s evaluation of search filters for finding articles in Medline [26] showed variation of 100 knowledge translation terms used, with only 46 of the 100 terms appearing in titles and abstracts of 500 articles, making them difficult to find. McKibbon *et al*. determined that many irrelevant articles were retrieved by knowledge translation search strategies, meaning that it is necessary to do a great deal of manual screening. Upon reviewing full document versions of potentially relevant papers, 52 relevant papers were included. Figure 1 shows the flow of work processes from database selection through to screening processes and the final selection of included papers.

Data were extracted from articles using bespoke forms, which were developed based on the content of the initial program theories. Data from each paper were extracted by two team members who cross-referred on decisions about content and relevance. Consistent with realist review evaluations of quality and decisions about research that was ‘good and relevant enough’ [20] to include were made during data extraction through the inclusion of a subjective evaluation of quality by the reviewer, with comments as to strengths and weaknesses of the study. The reviewers used the term ‘good and relevant enough’ to describe papers that provided detail on how conclusions were reached, without assessing quality in relation to study design or standardized criteria. We were also interested in fulfilling this criterion in relation to a study’s potential to add to the theoretical area. Good quality evidence included in-depth critical review [27] and incorporating thorough search methods [28], with data systematically reviewed and analyzed [29].

### Analysis and synthesis process

The investigators met for a face-to-face meeting and commenced analysis of the data collected in the extraction forms. The investigatory team divided into three subgroups to conduct the theming of the data, with each subgroup theming the extracted data according to one of the three research questions. Subgroup members independently themed the data extracted from each article using the question assigned to their subgroup. The subgroup then collated the themes identified by each of the members. From there, the subgroup members identified ‘chains of inference’ [12,25]. A chain of inference is a connection that can be made across articles based on the themes identified [23]. To establish a chain of inference, the theme must be evident in more than one paper. Subgroup members each shared the chains of inference they had identified. A conference call was then used to discuss, amend and/or confirm the chains of inference that had been proposed. To create an audit trail, for each chain of inference, articles containing themes that linked to individual chains of inference were recorded.

A second face-to-face meeting was then held to identify connections between the chains of inference and their effect on evidence-informed healthcare. Having articulated the connections, the group formulated hypotheses regarding the chains of inference. A chain of inference was, therefore, linked to each hypothesis and for each chain of inference themes from the literature were also linked. Further, all papers from which the themes related to the respective chains of inference were drawn, were clearly identified. Additional file 2 presents an audit trail of the stages of data extraction and how these stages inform the final set of themes arrived at, as shown in Figure 1 and summarized in this findings section.

### Main findings

**Figure 2:**
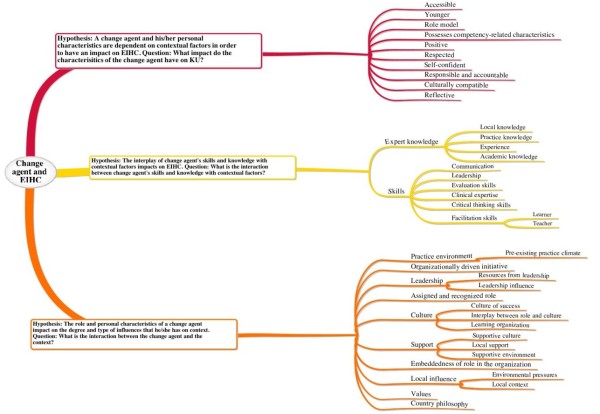
Themes derived from the data extraction linked to each of the research questions.

In order to be consistent with the theoretical framework that guided data extraction and analysis, the main findings are presented in response to each of the research questions posed for the change agency theory area. Figure 2 presents a diagrammatic representation of the themes derived from the data extraction linked to each of the research questions.

### How do the characteristics of the change agent affect knowledge utilization?

The literature provides some support for the potential of opinion leaders [30-38] and facilitators or facilitation [39-45] approaches to change agency, with a number of key features of change agents supported in this work to date. Such features include how responsibility and accountability [46,47] are established in a role, as well as how facilitators perceive the importance of their responsibility and accountability. Establishing respect as a change agent is also seen as crucial [32,48,49]. Beyond establishing respect, change agents must be role models of the evidence-informed healthcare values and practices they espouse [31,33,50,51].

Other features of changes agents identified in the literature but considered to be less important than those of responsibility, accountability, respect and role-modeling, include the age of the change agent (*e*.*g*., younger nurses have been associated with being able to influence higher levels of research use) [28], accessibility of the change agent (knowledge is more likely to be used when the change agent is perceived to be accessible, organized, expert and credible) [52], culturally compatible (perceived connection with the target group) [35,53], reflective [33,50,54], and having a positive attitude [28,33,46,48,54,55].

However, it is not clear from the evidence how these personal characteristics compare to one another in terms of their relative importance. There does seem to be some agreement that establishing respect and credibility, being positive, being a role model, and engaging in reflective practice are key features of a successful change agent. There is less evidence to support other personal characteristics found to be important, including accessibility, youth, responsibility/accountability, and cultural compatibility. Given that change agents engage in interpersonal activities, it can be assumed that personal characteristics are key to success, and the idea that change agency introduces the personal characteristics of the change agent as another ‘variable’ in the intervention has been a common criticism of evaluations of the approach [56]. However, the literature does not systematically evaluate or consistently comment on personal characteristics, and little has been done to evaluate how a change agent’s personal characteristics affect outcomes.

### How does the interaction between the change agent and the setting effect knowledge utilization?

The data did not produce evidence of specific effects of change agents on particular aspects of evidence use, but instead demonstrates the effect of change agents on creating the conditions for evidence-informed healthcare. We found that differences exist in published literature relating to the significance placed on ‘context’ in the reporting of studies in evidence-informed healthcare. Details of the intervention relating to the context for change agents’ work were not consistently provided. Seven papers failed to address context at all [28,36,49,51,57-59]. A further three papers provided some data on context but were either too limited in detail to allow inferences to be made [60,61], or the influence of context was alluded to but, based on the paper, it was not feasible to make any statements about which factors influenced effectiveness, or in which settings [62]. Despite inconsistency in reporting, from the studies that do consider contextual issues, some key issues emerge.

Consistent with the relationship between context and culture identified by Kitson *et al*. [56], our evidence provides support for the importance of the role of leadership and a supportive culture [41,63-65] as important components of context. The importance and significance of leaders actively supporting the use of evidence in practice as well as facilitating the creation of the conditions for evidence to be used was highlighted. Establishing a supportive culture appears to be important [50] and requires the removal of contextual and resource constraints [41]. The importance of local support [30,41,44] is emphasized. Additionally, a change agent who has a positive attitude, with well-established respect and credibility, seems more likely to be able to build a critical mass of leadership influence, solicit the required resources from leadership, and establish the necessary supportive culture. These aspects of the contexts in which change agents work seem to correspond to the personal characteristics of change agents that are supported by evidence.

The setting in which the implementation activity occurred was considered to play a key role in change agent success by authors of studies we reviewed. The characteristics of the setting that are given most consideration include: local influence (the extent to which the change agent is familiar with micro and meso influences in particular settings, and the extent to which they are able to influence these) [33,46] and culture [32,48,54,64]. Organizational culture was identified as a key issue in change agent success, particularly with respect to the interplay between change agent role and culture in a particular setting. However, the extent to which a change agent role is embedded in an organization is considered important in working with organizational culture and overcoming setting specific characteristics. The degree of embeddedness of a change agent was demonstrated in the literature to be an important contextual factor, with integration of the role into the organization being key [46,66,67]. While change agent roles that are embedded in an organization appear to be favored in the literature (as compared with external consultancy roles), caution is extended in the same literature with respect to loss of direction and confidence, unexpected pressures of work, high turnover due to poaching, and unrealistic expectations on the parts of the leaders themselves [46,48,63]. Within organizations, the change agent role needs to be viewed as important and adequately supported and resourced.

### What is the overall effect of the change agent on knowledge utilization?

While the data from this review provides insights into the conditions necessary for change agents to have an effect on evidence-informed healthcare, the data do not provide evidence of how particular mechanisms perform in certain contexts (settings). Positive personal characteristics appear to allow the change agent to capitalize on those aspects of the setting that can be influenced. Leadership and supportive culture seem to be intertwined with, or perhaps their effect can be strengthened by, the personal characteristics of respect, positivity, accessibility and responsibility/accountability. It seems likely that youth and cultural compatibility may affect a setting when there is a ‘fit’ between these characteristics and the setting in which the evidence is being implemented; *i*.*e*., there is a match between the age and cultural background of the change agent and the clinicians in the particular setting.

Our review suggests that change agents who are adequately supported and resourced (context) and who model the roles and practices they espouse (mechanism), have greater potential to achieve evidence-informed healthcare (outcome). These key findings can be summarized in the form of a Context (C), Mechanism (M), Outcome (O) configuration:

### Individual change agent and organizational contextual characteristics

A change agent needs to be embedded in the context and be accessible and organized. The change agent needs to be culturally compatible in terms of their established connections with the target group, be perceived by others as having expertise and as being credible. Change agents need to be clear about their lines of responsibility and accountability. Finally, in order to be effective, change agents need to operate in organizations that are supportive, where the role is seen as important and is adequately resourced.

### Mechanisms

Working with these contextual characteristics, a change agent needs to establish respect within the target group, display a positive attitude, act as a role model for the espoused practices, and show leadership.

### Outcome

A critical mass of leadership influence, soliciting of the required resources from leadership, and establishing the necessary supportive environment for change.

## Discussion

Given the potential impact of social interaction and face-to-face communication, change agent interventions and settings that capitalize on opportunities to facilitate interaction may be more successful, particularly in settings that feature a positive or receptive culture, a high degree of embeddedness, strong leadership support, and a good pre-existing local context. The literature did not offer clarity about how the role of leadership and having a supportive culture interact as part of context with change agents’ personal characteristics. The extent to which context, including setting, has been documented is highly varied within the current literature. The papers reviewed comment on particularly notable features of context and settings rather than systematically responding to a consistent set of features under consideration. This finding has been recently highlighted by Rycroft-Malone and colleagues in their implementation trial focusing on reducing pre-operative fasting times [8]. The study highlighted the multifactorial nature of context as well as a continued lack of clarity about what is meant by ‘context’. Chaudoir, Dugan and Barr highlight the need for agreement about the constructs that influence implementation success [68]. Consistent with other authors, while highlighting significant contextual features affecting implementation studies, our review did not add clarity to the range of contextual features that need to be considered. It is clear that a number of tangible and more abstract organizational/cultural factors are at play to moderate the effect of the context within which change agency is implemented, with some of these factors being considerably more difficult to manipulate and measure. For example, while leadership is a consistent feature of many of the studies reviewed, the specific qualities of leaders and how they enable evidence use in healthcare settings is poorly articulated and inconsistently applied. Research by Stetler *et al*. suggests that leaders (at every level) act as the ‘holders’ of an organization’s values and as such are pivotal to the way in which an evidence culture is promoted and operationalized [69]. This highlights the potential overlap between the mechanisms of action of change agents and leaders, which would be worth exploring further in the future, as would research into the ways in which leadership characteristics enable an organization’s values to be translated into meaningful action.

Additional difficulty arises from use in the literature of various terms relating to change agency in evidence-informed healthcare. In some cases, terms are used interchangeably and are not defined; for example, authors discuss the role of facilitator and also facilitation, and it is unclear whether there is any distinction. We caution that the term ‘practice development’ has contextual meanings, where there may well be other terms that hold implicit meanings in particular context(s), and this disclaimer may apply to other roles/terms as well. In general, the literature demonstrates careless use of language. It would be useful to be consistent, and if not, at least clear, with the use of terms in future publications. However, it would seem that there is still a long way to go in achieving this desired outcome, for example Flottorp and colleagues identified a taxonomy of 57 potential determinants of factors that prevent or enable improvements in healthcare professional practice [70].

A number of key features of the setting do appear to be facilitative (tangible resources, structures that enable change agency and intervention) and have potential for modification, with some less tangible features (quality of cultural characteristics, values) being more difficult to operationalize. In terms of forming partnerships and new relationships, change agents who are well-respected and easily able to act as role models may be more effective if they are able to interact effectively with individuals and teams. The potential of change agents to work within individuals and teams, including across professional and physical boundaries, seems to be a condition upon which people may be [more] successful in these roles [8]. Given the emerging importance of reflective practice, along with role modeling, a respected change agent who is a ‘fit’ in terms of their (cultural) compatibility may be more likely to succeed, and may also have a positive impact on the setting and thus, the outcome of interventions. Responsibility/accountability as important personal characteristics may also be a facilitator of local influence and help to generate a more receptive culture.

This knowledge and skill set assists change agents in addressing the variety of contextual issues that need to be addressed in bringing about change. In addition, these contextual issues can interact with personal characteristics and may impact the relative importance of particular characteristics and skills. The change agent role does not seem to require a formal position or formal authority, with social influence and social interaction being key components of the role. However, this is in contrast with the need for formal recognition of the role, and the potential for formality to facilitate influence over knowledge and information flow, an important part of change agency.

### Limitations

The limitations of this study relate to the review period, the quality of included papers, the sensitivity of the search strategy, as well as issues related to the lack of theory in published papers.

This realist review was undertaken in 2007 and included literature between the period of 1997 to 2007. This 10-year timespan includes a period of time when a large quantity of evidence-informed practice, knowledge translation and research utilization papers were published, and indeed represents the period when implementation science research began to emerge. However, we are conscious that in the six years since the review has been conducted, many more papers have been published that will add further to this review. While we are aware that our review adopted a focus on change agency as an intervention, we are confident that the literature included is representative of the research in this field at that time and that this realist review provides a platform for further development and expansion. We are not aware of any other review that has focused on ‘change agency’ as a concept or term in and of itself to date.

Included papers ranged from less-detailed accounts of ‘success stories’ [55] to those that described appropriate research designs, applied rigorous analysis and provided detailed presentation of findings [28]. We did not exclude papers based on study design or level of detail/evidence provided. Over half of the papers were assessed as contributing to an understanding of change agency in knowledge utilization for evidence-informed healthcare. Five papers [33,46,49,54,71] provided detailed accounts of original research with sufficient supplementary detail to contribute to a clear picture of the research process and intervention. These papers used appropriate research designs for the questions addressed, reported rigorous analyses and detailed findings, clearly describing the methodology of the project, the intervention itself, and any evaluation undertaken. However, detail on either methods or the intervention itself was lacking in 10 papers [31,35,45,47,48,51,55,62],[72-74]. A great deal can be learned from papers in which sufficient detail is provided to assess whether claims made are supported in the data [12,27-29,33,46,49,54,64,71].

Use of well-established theories was not uniformly evident across included papers. In fact, absence of theory was almost as frequent as use of a theoretical framework; and reference to individual concepts, such as research utilization [75], facilitation or practice development was also apparent [27,41,76]. More specifically, many papers did not explicitly discuss the theoretical underpinnings supporting development, implementation or evaluation of change agent interventions. Some of the papers that did refer to a theory, or on occasion more than one theory, merely noted use in terms of framing or informing a project related to a change agent, for example, through use of a variety of learning theories, including Knowles’ adult learning theory [77], critical reflection [78], and forms of knowing [79-81]. Other theoretically-based studies used a variety of theories to design their intervention (social cognitive theory [53], social marketing [38], diffusion of innovations [35] and Promoting Action on Research Implementation in Health Services [PARIHS] [43]). The predominant framework underpinning this work was Rogers’ Diffusion of Innovation Theory. Overall, theory appeared to be underutilized and not uniformly described in sufficient detail for one to fully understand its role in the study of change agents.

Finally, while we have attempted to make our search strategy as sensitive as possible (and erred on the side of sensitivity as opposed to specificity), we are conscious that Knowledge Utilization continues to be a poorly indexed area of literature, and so it is difficult to design a perfect search strategy. In order to compensate for this limitation, we involved the entire group in overseeing and reviewing search strategies and search results in order to ensure we were being as comprehensive as possible.

## Conclusions

As the first realist review of change agency research, a comprehensive, inclusive review of the published evidence has been produced, summarizing what the literature demonstrates about the personal characteristics and context within which change agency functions as well as the effectiveness of change agents. The review highlights significant gaps and provides direction for future development of change agency for evidence-informed healthcare. Change agency strategies currently used to foster knowledge utilization include opinion leaders, facilitators (internal/external), practice developers, education outreach, academic detailing, and the use of multiple change agents. While evidence of effectiveness is weak, in some cases in terms of outcomes data, there is evidence that supports the importance of opinion leader and facilitator roles. The literature would benefit from better descriptions of interventions and determination of outcomes, as well as more detail on the context, intensity and levels at which interventions are implemented. If a focus on measurement of appropriate outcomes and detailed reporting can be realized, the field can learn more from the implementation of change agency roles across different contexts. At this time, there does not seem to be adequate evidence to assess whether particular roles and associated mechanisms are more effective in particular contexts, or to make generalizations about which change agent(s) work best for which professional groups or settings. However, there is emerging evidence to suggest that ‘fit’ might be an important mechanism, such that if there is compatibility between a change agent’s characteristics, approach, relationship with individuals and teams, and the contextual conditions in which they are working, their chances of being more successful in change agency might be enhanced. This review contributes to what we know about contexts in which the effect of change agency can occur, mechanisms through which change agency functions, and outcomes addressed in the literature within the scope of this review. It is expected that additional work to test hypotheses generated could further contribute to what is known about the contexts, mechanisms, and outcomes related to change agency effectiveness.

## Competing interests

Alison Hutchinson is a member of the International Editorial Board of Implementation Science. Bridie Kent is an Associate Editor for Implementation Science; other editors made all decisions on this manuscript.

## Authors’ contributions

BM and JRM led the project. All authors participated in defining the scope of the review. KD and ESC executed the search. All authors undertook the appraisal of evidence and data extraction. All authors were involved in the analysis process. AMH led the documentation of the study process. KD led the development of the narrative. BM wrote the first draft of the paper; JRM, AH, and KD commented on it. All authors provided feedback on various drafts, and read and approved the final manuscript.

## Supplementary Material

Additional file 1 Search strategies and databases. Click here for file

Additional file 2 seven‒step approach to data analysis and synthesis. Click here for file
