# Supplementary material for: A realist review of interventions and strategies to promote evidence-informed healthcare: a focus on change agency
Source: Implement Sci. 2013 Sep 8;8:107. doi: 10.1186/1748-5908-8-107 (PMC3848622; doi:10.1186/1748-5908-8-107)
Supplement: Additional file 1 — Search strategies and databases. [file 1748-5908-8-107-S1.pdf]

Additional File 1

Search Strategies and Databases

Electronic Search Strategies

Search Strategies for Medline, Embase, CINAHL, PsycInfo, Sociological Abstracts, Web of Science

Executed March 7 – March 9, 2007

Database: Ovid MEDLINE(R)

Search Strategy:

----

1 opinion leader.mp. [mp=title, original title, abstract, name of substance word, subject heading word] (44)

2 facilitat$.mp. [mp=title, original title, abstract, name of substance word, subject heading word] (147970)

3 education outreach$.mp. [mp=title, original title, abstract, name of substance word, subject heading word] (30)

4 academic detail$.mp. [mp=title, original title, abstract, name of substance word, subject heading word] (154)

5 practice develop$.mp. [mp=title, original title, abstract, name of substance word, subject heading word] (439)

6 clinical educator.mp. [mp=title, original title, abstract, name of substance word, subject heading word] (27)

7 change agen$.mp. [mp=title, original title, abstract, name of substance word, subject heading word] (314)

8 knowledge broker$.mp. [mp=title, original title, abstract, name of substance word, subject heading word] (9)

9 champion.mp. [mp=title, original title, abstract, name of substance word, subject heading word] (438)

10 innovator.mp. [mp=title, original title, abstract, name of substance word, subject heading word] (245)

11 boundary spann$.mp. [mp=title, original title, abstract, name of substance word, subject heading word] (25)

12 advoca$.mp. [mp=title, original title, abstract, name of substance word, subject heading word] (43602)

13 expert.mp. [mp=title, original title, abstract, name of substance word, subject heading word] (24830)

14 transformational leader$.mp. [mp=title, original title, abstract, name of substance word, subject heading word] (155)

15 consultan$.mp. [mp=title, original title, abstract, name of substance word, subject heading word] (12050)

16 exp *Consultants/ (2344)

17 coach$.mp. [mp=title, original title, abstract, name of substance word, subject heading word] (2663)

18 educator.mp. [mp=title, original title, abstract, name of substance word, subject heading word] (1755)

19 exp *Health Educators/ (39)

20 1 or 2 or 3 or 4 or 5 or 6 or 7 or 8 or 9 or 10 or 11 or 12 or 13 or 14 or 15 or 16 or 17 or 18 or 19 (230837)

21 knowledge transfer.mp. [mp=title, original title, abstract, name of substance word, subject heading word] (140)

22 knowledge translation.mp. [mp=title, original title, abstract, name of substance word, subject heading word] (63)

23 knowledge uptake.mp. [mp=title, original title, abstract, name of substance word, subject heading word] (11)

24 knowledge utilization.mp. [mp=title, original title, abstract, name of substance word, subject heading word] (38)

25 knowledge exchange.mp. [mp=title, original title, abstract, name of substance word, subject heading word] (36)

26 research uptake.mp. [mp=title, original title, abstract, name of substance word, subject heading word] (8)

27 research utilization.mp. [mp=title, original title, abstract, name of substance word, subject heading word] (346)

28 knowledge utilisation.mp. [mp=title, original title, abstract, name of substance word, subject heading word] (3)

29 research utilisation.mp. [mp=title, original title, abstract, name of substance word, subject heading word] (21)

30 evidence based$.mp. [mp=title, original title, abstract, name of substance word, subject heading word] (31693)

31 evidence informed$.mp. [mp=title, original title, abstract, name of substance word, subject heading word] (29)

32 innovation.mp. [mp=title, original title, abstract, name of substance word, subject heading word] (25045)

33 research use$.mp. [mp=title, original title, abstract, name of substance word, subject heading word] (615)

34 research translation.mp. [mp=title, original title, abstract, name of substance word, subject heading word] (8)

35 research trans$.mp. [mp=title, original title, abstract, name of substance word, subject heading word] (79)

36 exp *Organizational Innovation/ or exp *"Diffusion of Innovation"/ (6981)

37 21 or 22 or 23 or 24 or 25 or 26 or 27 or 28 or 29 or 30 or 31 or 32 or 33 or 34 or 35 or 36 (57602)

38 20 and 37 (4530)

39 from 38 keep 1-1000 (1000)

****

Database: EMBASE

Search Strategy:

----

1 opinion leader.mp. (42)

2 facilitat$.mp. (135598)

3 education outreach$.mp. (23)

4 exp *medical education/ (34690)

5 academic detail$.mp. (155)

6 practice develop$.mp. (163)

7 exp *Clinical Education/ or clinical educator.mp. (2010)

8 change agen$.mp. (158)

9 knowledge broker$.mp. (6)

10 champion.mp. (259)

11 innovat$.mp. (20668)

12 boundary spann$.mp. [mp=title, abstract, subject headings, heading word, drug trade name, original title, device manufacturer, drug manufacturer name] (17)

13 advoca$.mp. [mp=title, abstract, subject headings, heading word, drug trade name, original title, device manufacturer, drug manufacturer name] (19519)

14 expert.mp. or *EXPERT SYSTEM/ or *MEDICAL EXPERT/ or *EXPERT NURSE/ (21317)

15 *Leadership/ or transformational leader$.mp. (1010)

16 consultan$.mp. [mp=title, abstract, subject headings, heading word, drug trade name, original title, device manufacturer, drug manufacturer name] (7935)

17 coach$.mp. (2234)

18 *HEALTH EDUCATOR/ or *DIABETES EDUCATOR/ or educator.mp. (942)

19 1 or 2 or 3 or 4 or 5 or 6 or 7 or 8 or 9 or 10 or 11 or 12 or 13 or 14 or 15 or 16 or 17 or 18 (237986)

20 knowledge trans$.mp. (197)

21 knowledge transfer.mp. (115)

22 knowledge translation.mp. (47)

23 knowledge uptake.mp. (8)

24 knowledge utilization.mp. (13)

25 knowledge exchange.mp. (19)

26 research uptake.mp. (4)

27 research utilization.mp. (55)

28 research utilisation.mp. (11)

29 knowledge utilisation.mp. (3)

30 evidence-based$.mp. or exp *Evidence Based Practice/ (40261)

31 exp *EVIDENCE BASED NURSING/ or exp *EVIDENCE BASED PRACTICE CENTER/ or exp *EVIDENCE BASED MEDICINE/ (7320)

32 innovation.mp. (4209)

33 exp *NURSING EVALUATION RESEARCH/ or exp *MEDICAL RESEARCH/ or exp *RESEARCH UTILIZATION GROUP/ or exp *NURSING RESEARCH/ or exp *RESEARCH/ (39951)

34 research trans$.mp. (62)

35 organizational innovation.mp. (21)

36 20 or 21 or 22 or 23 or 24 or 25 or 26 or 27 or 28 or 29 or 30 or 31 or 32 or 33 or 34 or 35 (82955)

37 19 and 36 (10248)

38 limit 37 to human (8482)

39 from 38 keep 1-1000 (1000)

****

Database: CINAHL - Cumulative Index to Nursing & Allied Health Literature <1982 to February Week 4 2007>

Search Strategy:

----

1 opinion leader.mp. [mp=title, subject heading word, abstract, instrumentation] (18)

2 facilitat$.mp. [mp=title, subject heading word, abstract, instrumentation] (15597)

3 education outreach$.mp. [mp=title, subject heading word, abstract, instrumentation] (20)

4 academic detail$.mp. [mp=title, subject heading word, abstract, instrumentation] (46)

5 practice develop$.mp. [mp=title, subject heading word, abstract, instrumentation] (3397)

6 clinical educator.mp. [mp=title, subject heading word, abstract, instrumentation] (41)

7 exp Clinical Nurse Specialists/ or clinical nurse.mp. (6609)

8 change agen$.mp. [mp=title, subject heading word, abstract, instrumentation] (425)

9 knowledge broker$.mp. [mp=title, subject heading word, abstract, instrumentation] (4)

10 champion.mp. (288)

11 innovat$.mp. [mp=title, subject heading word, abstract, instrumentation] (9855)

12 boundary spann$.mp. [mp=title, subject heading word, abstract, instrumentation] (15)

13 advoca$.mp. [mp=title, subject heading word, abstract, instrumentation] (12804)

14 expert.mp. [mp=title, subject heading word, abstract, instrumentation] (6522)

15 exp EXPERT NURSES/ or exp EXPERT CLINICIANS/ or exp EXPERT SYSTEMS/ (1388)

16 exp Nursing Leaders/ or exp Leadership/ or transformational leader$.mp. (10192)

17 consultan$.mp. [mp=title, subject heading word, abstract, instrumentation] (5086)

18 coach$.mp. (1473)

19 educator.mp. or exp HEALTH EDUCATORS/ or exp DIABETES EDUCATORS/ or exp CHILDBIRTH EDUCATORS/ (3112)

20 knowledge trans$.mp. (657)

21 knowledge uptake.mp. (6)

22 exp Nursing Knowledge/ or exp Knowledge/ or knowledge utilization.mp. (12571)

23 knowledge exchange.mp. (31)

24 exp MEDICAL PRACTICE, RESEARCH-BASED/ or exp NURSING PRACTICE, RESEARCH-BASED/ or exp HEALTH SERVICES RESEARCH/ or exp PROFESSIONAL PRACTICE, RESEARCH-BASED/ or exp PHYSICAL THERAPY PRACTICE, RESEARCH-BASED/ (9511)

25 research uptake.mp. (58)

26 research utilization.mp. (1830)

27 research utilisation.mp. (48)

28 knowledge utilisation.mp. (1)

29 evidence based.mp. (14454)

30 evidence informed.mp. (16)

31 research trans$.mp. [mp=title, subject heading word, abstract, instrumentation] (4239)

32 organizational innovation.mp. [mp=title, subject heading word, abstract, instrumentation] (216)

33 exp "DIFFUSION OF INNOVATION"/ (1164)

34 1 or 2 or 3 or 4 or 5 or 6 or 7 or 8 or 9 or 10 or 11 or 12 or 13 or 14 or 15 or 16 or 17 or 18 or 19 (69431)

35 20 or 21 or 22 or 23 or 24 or 25 or 26 or 27 or 28 or 29 or 30 or 31 or 32 or 33 (40961)

36 34 and 35 (5638)

37 from 36 keep 1-1000 (1000)

Database: PsycINFO

Search Strategy:

----

1 opinion leader.mp. (24)

2 facilitat$.mp. [mp=title, abstract, heading word, table of contents, key concepts] (41811)

3 education outreach$.mp. [mp=title, abstract, heading word, table of contents, key concepts] (16)

4 academic detail$.mp. [mp=title, abstract, heading word, table of contents, key concepts] (32)

5 practice develop$.mp. (209)

6 clinical educator.mp. (10)

7 change agen$.mp. [mp=title, abstract, heading word, table of contents, key concepts] (618)

8 knowledge broker$.mp. (11)

9 champion.mp. (226)

10 innovat$.mp. [mp=title, abstract, heading word, table of contents, key concepts] (12735)

11 innovat$.mp. [mp=title, abstract, heading word, table of contents, key concepts] (12735)

12 boundary spann$.mp. [mp=title, abstract, heading word, table of contents, key concepts] (150)

13 advoca$.mp. [mp=title, abstract, heading word, table of contents, key concepts] (12409)

14 expert.mp. (11444)

15 exp Transformational Leadership/ or transformational leader$.mp. (793)

16 consultan$.mp. [mp=title, abstract, heading word, table of contents, key concepts] (4989)

17 coach$.mp. [mp=title, abstract, heading word, table of contents, key concepts] (3579)

18 educator.mp. (1538)

19 1 or 2 or 3 or 4 or 5 or 6 or 7 or 8 or 9 or 10 or 11 or 12 or 13 or 14 or 15 or 16 or 17 or 18 (86102)

20 knowledge trans$.mp. (427)

21 knowledge uptake.mp. (4)

22 knowledge utilization.mp. (55)

23 knowledge utilisation.mp. (0)

24 research uptake.mp. (2)

25 exp Evidence Based Practice/ or evidence based$.mp. (5371)

26 evidence informed$.mp. (23)

27 innovation.mp. (4051)

28 research use$.mp. (706)

29 research trans$.mp. (56)

30 organizational innovation.mp. (104)

31 organisational innovation.mp. (2)

32 diffusion of innovation.mp. (74)

33 20 or 21 or 22 or 24 or 25 or 26 or 27 or 28 or 29 or 30 or 31 or 32 (10556)

34 19 and 33 (4993)

35 from 34 keep 1-1000 (1000)

****

Search Strategy

Web of Science

March 9, 2007

(TI=knowledge trans* OR TI=knowledge utili?ation OR TI=knowledge exchange OR TI=knowledge uptake OR TI=knowledge use OR TI=research trans* OR TI=research utili?ation OR TI=research uptake OR TI=research use OR TI=evidence based OR TI=evidence informed OR TI=innovation OR TI=organi?ational innovation OR TI=diffusion of innovation) AND (TI=opinion leader OR TI=facilitator OR TI=facilitation OR TI=education outreach work* OR TI=academic detail* OR TI=practice develop* OR TI=clinical educator OR TI=change agen* OR TI=knowledge broker OR TI=knowledge brokers OR TI=knowledge brokering OR TI=champion OR TI=innovator OR TI=boundary spann* OR TI=advoca* OR TI=expert OR TI=transformational leader OR TI=transformational leader* OR TI=consultant OR TI=consultan* OR TI=coach OR TI=educator)

CSA

Multiple Databases

Query: (((DE=("information dissemination" or "diffusion" or "knowledge utilization")) and (DE="evidence based practice") and (DE=("diffusion" or "adoption of innovations")) and (DE=("innovations" or "technological innovations"))) or (((knowledge trans*) or (knowledge uptake) or (knowledge use)) or ((knowledge utili?ation) or (knowledge exchange) or (research trans*)) or ((research uptake) or (research use) or (research utili?ation)) or ((evidence base*) or (evidence informed) or innovation) or ((organi?ational innovation) or (diffusion of innovation)))) and (((opinion leaders) or (opinion leader) or facilitat*) or ((education outreach worker) or (academic detail*) or (practice develop*)) or ((clinical educator) or (change agents) or (change agent)) or ((change agency) or (knowledge brok*) or champion) or (innovator or (boundary spann*) or advocacy) or (advocate or experts or expert) or

((transformational lead*) or consultants or consultant))

Your Comments: soc abstracts results

Hand Searched Journals

Administrative Science Quarterly

Canadian Journal of Nursing Research

Image: The Journal of Nursing Scholarship

Knowledge: Creation, Diffusion, Utilization

Knowledge and Policy

Nursing Science Quarterly

Nursing Research

Worldviews in EBN

Journal of Advanced Nursing

British Medical Journal

Advances in Nursing Science

Evidence Based Nursing

Science Communication

Knowledge and Policy
