# Supplementary material for: A realist review of interventions and strategies to promote evidence-informed healthcare: a focus on change agency
Source: Implement Sci. 2013 Sep 8;8:107. doi: 10.1186/1748-5908-8-107 (PMC3848622; doi:10.1186/1748-5908-8-107)
Supplement: Additional file 2 — seven‒step approach to data analysis and synthesis. [file 1748-5908-8-107-S2.pdf]

Additional File 2:

Seven-step approach to data analysis and synthesis

(Data Analysis) Step 1: organising the data into evidence-tables

**THEORY AREA 1 - What impact do the characteristics of the change agent have on KU?**	
**Moore, KA; Peters, RH; Hills, HA; Levasseur, JB; Rich, AR; Hunt, WM; Young, MS, Valente, TW (2004)**	The study identified a number of 'competency – related' characteristics of opinion leaders (OL) that are significant, including – postgraduate education; relevant professional credentials; years of experience in the treatment area. Identified opinion leaders (n=10) were compared with other counsellors (n=55).<br>In this study, OLs were found to have more postgraduate education, more relevant professional credentials and more postgraduate qualifications.<br>"OLs had significantly more work experience in mental health than their co-workers, and were twice as likely to have postgraduate education in comparison to their peers … OLs were more confident and willing to work with clients who have co-occurring disorders. In comparison to their colleagues, OLs had significantly greater knowledge regarding diagnosis and treatment of co-occurring disorders" (p199)
**Stanley D (2006)**	This paper does not specifically focus on KU but it is still helpful to KU with its focus on the characteristics of clinical leaders.<br>A clinical leader is defined as "one who possesses clinical expertise in a specialty practice area and who uses interpersonal skills to enable nurses and other healthcare providers to deliver quality patient care" (p108).<br>The study of the characteristic of clinical leaders suggests that "… it is the demonstration and translation of their values and beliefs into the actions and the functions of their role for which they are admired and followed" (p110).
**Mercier C; Bordeleau M; Caron J; Garcia A and Latimer E (2004)**	The change agents were a research team in a healthcare institution. Thus they were known to the participants and had awareness of the contextual factors.

(Data Analysis) Data Step 2 – identification of individual themes

Reviewer 1

**Moore, KA; Peters, RH; Hills, HA; Levasseur, JB; Rich, AR; Hunt, WM; Young, MS, Valente, TW (2004)**	The study identified a number of 'competency – related' characteristics of opinion leaders (OL) that are significant, including – postgraduate education; relevant professional credentials; years of experience in the treatment area. Identified opinion leaders (n=10) were compared with other counsellors (n=55).<br>In this study, OLs were found to have more postgraduate education, more relevant professional credentials and more postgraduate qualifications.<br>"OLs had significantly more work experience in mental health than their co-workers, and were twice as likely to have postgraduate education in comparison to their peers … OLs were more confident and willing to work with clients who have co-occurring disorders. In comparison to their colleagues, OLs had significantly greater knowledge regarding diagnosis and treatment of co-occurring disorders" (p199)	**More Postgraduate education, more postgraduate qualifications and more professional credentials were found among opinion leaders than other counsellors.**

Reviewer 2

**Moore, KA; Peters, RH; Hills, HA; Levasseur, JB; Rich, AR; Hunt, WM; Young, MS, Valente, TW (2004)**	The study identified a number of 'competency – related' characteristics of opinion leaders (OL) that are significant, including – postgraduate education; relevant professional credentials; years of experience in the treatment area. Identified opinion leaders (n=10) were compared with other counsellors (n=55).<br>In this study, OLs were found to have more postgraduate education, more relevant professional credentials and more postgraduate qualifications.<br>"OLs had significantly more work experience in mental health than their co-workers, and were twice as likely to have postgraduate education in comparison to their peers … OLs were more confident and willing to work with clients who have co-occurring disorders. In comparison to their colleagues, OLs had significantly greater knowledge regarding diagnosis and treatment of co-occurring disorders" (p199)	**Characteristics influencing practice knowledge/competence were:**<br>**Post graduate education**<br>**Professional credentials**<br>**Years of experience**<br>**Confidence**<br>**Willing to work with clients w/ co-disorders**

(Data Analysis) Steps 3 and 4 – merging of individual themes and identification of chains of inference

Author	Individual comments	Step 3. Amalgamated themes	Step 4: Look for chain of inferences (connections across papers) to ultimately develop hypotheses	Decision/recommendations of conference call
1) Moore, KA; Peters, RH; Hills, HA; Levasseur, JB; Rich, AR; Hunt, WM; Young, MS, Valente, TW (2004)	Does not address impact change agent characteristics on KU but does address characteristics of OL in practice/competence	Post graduate education.<br>Professional credentials.<br>Years of experience.<br>Confidence.<br>Greater knowledge.	Expert Knowledge<br>Professional qualifications<br>Experience<br>Positive attitude	Need to go back to article and determine if "confidence" is related to knowledge or experience;<br>Possibly remove positive attitude based on review of article

Step 5: Impact assessment

Identification/isolation of those papers that provided some evidence of impact in terms of evidence of "what worked, for whom, in what circumstances and why … in relation to EIHC

(Synthesis) Step 6: Chains of Inference

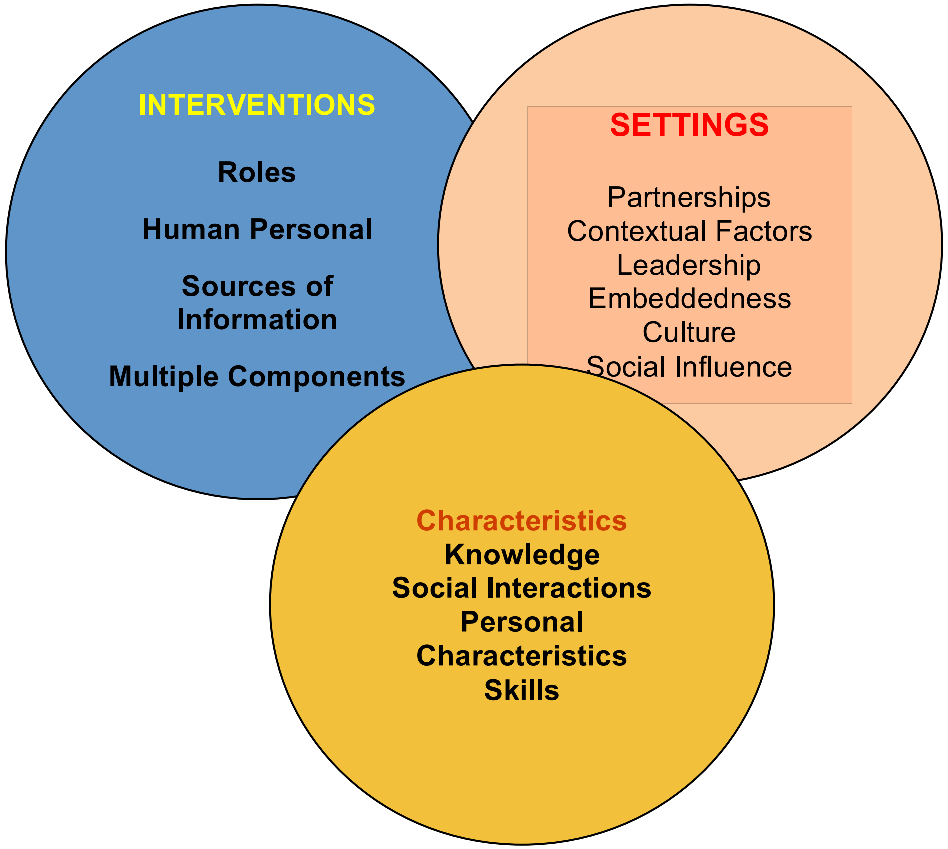

Themes within Chains of Inference

Chains of Inference	Derived from the following themes in step 3	Articles
**Knowledge**	**Professional qualifications<br>Expert knowledge<br>Knowledgeable<br>Local knowledge**	**1,3, 6,7,10, 11, 13, 14,15, 16,18,19,20,21, 22, 23, 25, 29,35,36,37,39**
**Skills**	**Communication skills<br>Leadership skills<br>Thinking skills<br>Clinical skills<br>Cognitive skills<br>Evaluation skills<br>Political skills<br>Facilitation Skills<br>Reflective skills**	**2, 4,5,6,7,8,9,10,11,12, 13, 14,15,16, 17, 18,19,20,21, 22, 24,25,27,28,32, 33, 34,36,38,39,40**
**Personal Characteristics**	**Role model<br>Positive attitude<br>Responsibility/accountability<br>Respected<br>Information Seeking<br>Positive Attitude<br>Accessible<br>Age<br>Teacher<br>Culturally compatible<br>Objectivity<br>Years of experience**	**1,2,4, 6,7,8, 13, 14, 15, 16, 17,18, 22,28,29,30,31,32,33,35,36, 37,38, 39**
**Social Interaction**	**Social Influence<br>Networking<br>Shared Ownership**	**5,8,12, 15,18,31,39,40, Wright**

Connections among Chains of Inference

- Nature of relationship …
  - between change agent (CA) personal characteristics and the ability to form partnerships on the impact of EIHC.
  - among CA personal characteristics, role adopted, and contextual influences on the impact of EIHC.
  - between CA personal characteristics, skills, and knowledge and the interplay with contextual influences on the impact of EIHC.
  - Between knowledge and human personal sources of information on the impact of EIHC.
  - among contextual influence, social interaction, CA personal characteristics and human personal sources of information and impact on the EIHC.
  - among partnership, social interaction, contextual influence, and skills on the impact of EIHC.
  - between skills and roles on the impact of EIHC.

(Synthesis) Step 7: Hypotheses Generation (example)

Hypotheses	Chain of Inference (theory level)	Chain of inference (sub-theory level)	Themes from the literature	Papers addressing the theme
An opinion leader and his/her personal characteristics are dependent on contextual factors in order to have an impact on EIHC.<br>A facilitator and his/her personal characteristics are dependent on contextual factors in order to have an impact on EIHC.	The nature of the relationship between the change agent's personal characteristics, the role adopted, and contextual influences and the impact of EIHC.	Roles<br>Personal Characteristics<br>Contextual Factors	Opinion Leader<br>Facilitator	*Papers with mixed and positive effects, only:*<br>6 OL (Wright, Chaillet, Curran, Moore, Davies, Majumdar)<br><br>6 FAC (int/ext and ext fac incl), (Stetler, Cranney, Gerrish, Milner, Thomas, Hutt)<br>Total 18 CA papers, 12 OL and FAC
